# Monitoring the Use of Telemonitor: A Resident-run Quality Improvement Initiative Decreases Inappropriate Use of Telemonitor in a Community Hospital

**DOI:** 10.7759/cureus.6263

**Published:** 2019-11-30

**Authors:** Tinashe Maduke, Binish Qureshi, Yohannes Goite, Khushboo Gandhi, Fadel Bofarrag, Lin Liu, Miguel Suazo, Sehrish Khan, Samjhana Basnyat, Suresh Dhital, Hameem Kawsar

**Affiliations:** 1 Internal Medicine, St. Luke's Hospital, Chesterfield, USA; 2 Internal Medicine/Hematology and Oncology, University of Kansas Medical Center, Kansas City, USA

**Keywords:** telemonitor use, telemetry, quality improvement, choosing wisely, patient safety

## Abstract

Background

Cardiac telemetry is an important tool to detect life-threatening conditions in hospitalized patients but is used widely and inappropriately. We sought to assess current usage and improve the appropriate use of telemetry in a community hospital.

Methods

We conducted a quality improvement project on patients who were admitted on telemetry floors between January and March 2017 (pre-intervention). The indication(s) and duration of telemonitor use, event(s) recorded on telemonitor and outcome of the event(s) were documented. A six-month educational intervention was undertaken and the effect of intervention was assessed among patients admitted between December 2017 and February 2018 (post-intervention).

Results

In the pre-intervention group, 329 patients qualified for the study, with a median age of 78 years. The post-intervention group had 383 qualified patients with a median age of 77 years. Mean duration of telemonitor use was four days in both groups. In the pre-intervention group, 54% had class I, 32% had class II, and 14% had class III indications. In post-intervention group, 46% had class I, 42% had class II, and 12% had class III indications. The educational intervention resulted in a trend towards less inappropriate use of telemetry, particularly in teaching service. Telemonitor events were recorded in 22 (7%) of the pre-intervention patients and 13 (4%) of the post-intervention group. Two patients died in the pre-intervention group and one in the post-intervention group from non-cardiac causes.

Conclusion

Our results highlight that change in practice requires sustained education interventions.

## Introduction

In 2004, American Heart Association (AHA) released guidelines on the use of telemonitor, and divided them into three classes: class I (telemonitor use indicated), class II (telemonitor may be beneficial) and class III (telemonitor not indicated) [1]. Studies have shown that telemonitor is often overused and used inappropriately. The Choosing Wisely® campaign, an American Board of Internal Medicine (ABIM) foundation initiative aimed at reducing waste and unnecessary tests in healthcare, recommended the use of guidelines to improve appropriate use of telemonitor in patients outside of ICU. Inappropriate use of telemetry has been widely studied and has shown to negatively affect patient care [2]. It can increase length of stay, cause alarm fatigue among providers, impact patient satisfaction, impact admission rates and cause backup in the emergency department [3, 4]. It also adds extra cost to healthcare delivery [5]. The objective of this study was to evaluate the effectiveness of an educational intervention in reducing the inappropriate use of telemetry.

## Materials and methods

Study design and sampling

We used a retrospective study looking at inpatient records of 712 patients in a 493-bed community teaching hospital in St Louis, MO. Inclusion criteria consisted non-ICU patients admitted on the telemetry floor or on a medical floor with remote cardiac monitoring. We excluded patients younger than 18 years of age, ICU patients, prior admission to ICU before transfer to telemetry floor, obstetrics and gynecology patients. We reviewed patients’ chart who used cardiac monitor between January and March 2017 (pre-intervention) and December 2017 to February 2018 (post intervention). We selected patients who had cardiac monitoring ordered on admission and during admission. The study was approved by the hospital’s institutional review board (IRB).

Data collection

We determined indications for cardiac monitoring from admission and progress notes. Duration on cardiac monitor was collected using order and cancellation dates and times from the electronic medical record. Data collection included principal diagnoses, age, gender, sex, days on telemetry, arrhythmias detected, rapid response/code blue events and transfer to ICU events. Data was gathered from St Luke’s hospital electronic medical record system (Cerner) and recorded and analyzed on a secure Microsoft Excel and IBM SPSS.

Educational intervention

The intervention involved educational sessions with the hospitalists, Internal Medicine residents, cardiologists, emergency department physicians and nurse practitioners on the AHA cardiac monitoring guideline criteria for when it is indicated, beneficial and where it is not indicated. Each hospitalist, nurse practitioner and resident also received pocket cards with the criteria listed for them to use as a quick reference to decide when to order telemetry. Educational sessions were conducted as small group conferences, and structured and predefined reminder sessions for all participants. These sessions were conducted from August to November, 2017 with multiple reminder/refresher sessions.

Outcomes

The primary outcome was appropriateness of use of telemetry monitor as determined by the 2004 AHA guidelines. Secondary outcomes included duration on telemetry, events detected on cardiac monitor, occurrence of significant clinical events and death.

Statistical analysis

Continuous variables were analyzed using t-tests and ANOVA. Categorical data was analyzed using chi-square tests.

## Results

Patient characteristics

**Figure 1:**
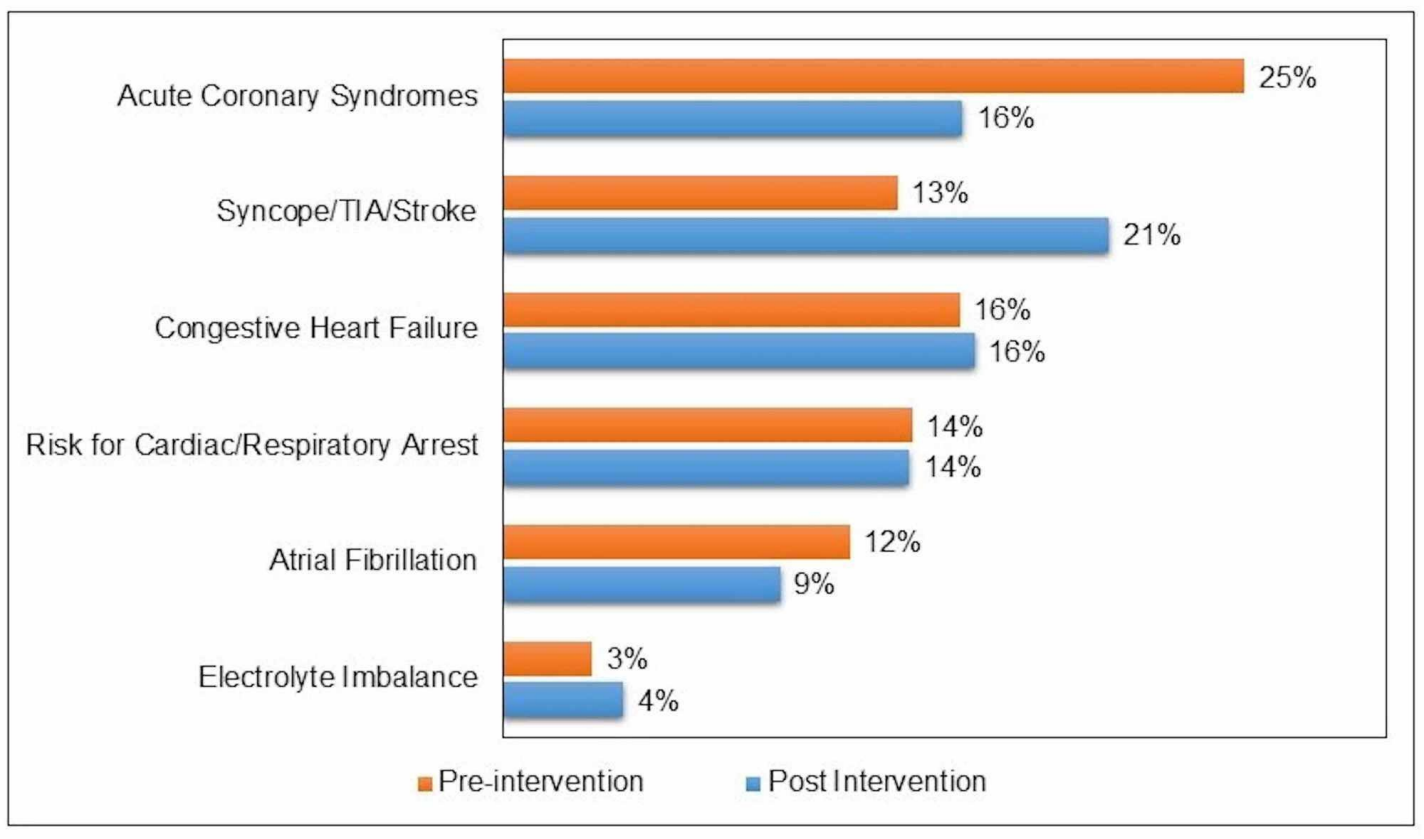
Common reasons for using telemetry.

In the pre-intervention group, 329 patients qualified for the study during January to March of 2017. Of these, 162 were in teaching service and 167 were in non-teaching service. The median age was 78 years and 48% of the patients were female. The data for post-intervention group was collected between December 2017 and February 2018, and 383 patients qualified for the study. The teaching service had 148 patients, while 245 patients were in non-teaching service. The median age was 77 years and 54% were female. Table 1 shows the characteristic of patients in both groups. The mean duration of telemonitor use was four days in both groups. Telemetry was continued until discharge in 93% of pre-intervention group and 91.6% of post intervention group (p = 0.52). The main reasons for telemetry use were acute coronary syndromes, syncope, stroke/transient ischemic attack, decompensated congestive heart failure, atrial fibrillation and electrolyte abnormalities (Figure 1).

**Table 1:** Patient and event characteristics.

	Pre-intervention	Post-intervention	p-value
Sample size	329	383	
Teaching/non-teaching	162/167	148/235	
Age (median, range)	78 (34-100)	77 (19-81)	0.901
Gender, female (%)	158 (48%)	208 (54.3%)	0.157
AHA classes of indication for telemetry use			
I	179 (54.4%)	176 (46.0%)	0.025
II	105 (31.9%)	162 (42.3%)	0.004
III	45 (13.7%)	45 (11.7%)	0.42
Days on cardiac monitor (mean)	4.03	4.05	0.928
Cardiac monitor used until discharge	300 (93.2%)	351 (91.6%)	0.518
Events on monitor	22 (6.7%)	14 (3.7%)	0.066
Actions taken for monitored event(s)	6 (27.3%)	4 (26.7%)	0.967
Deaths	2	1	0.476

Use of telemonitor according to AHA indications

**Figure 2:**
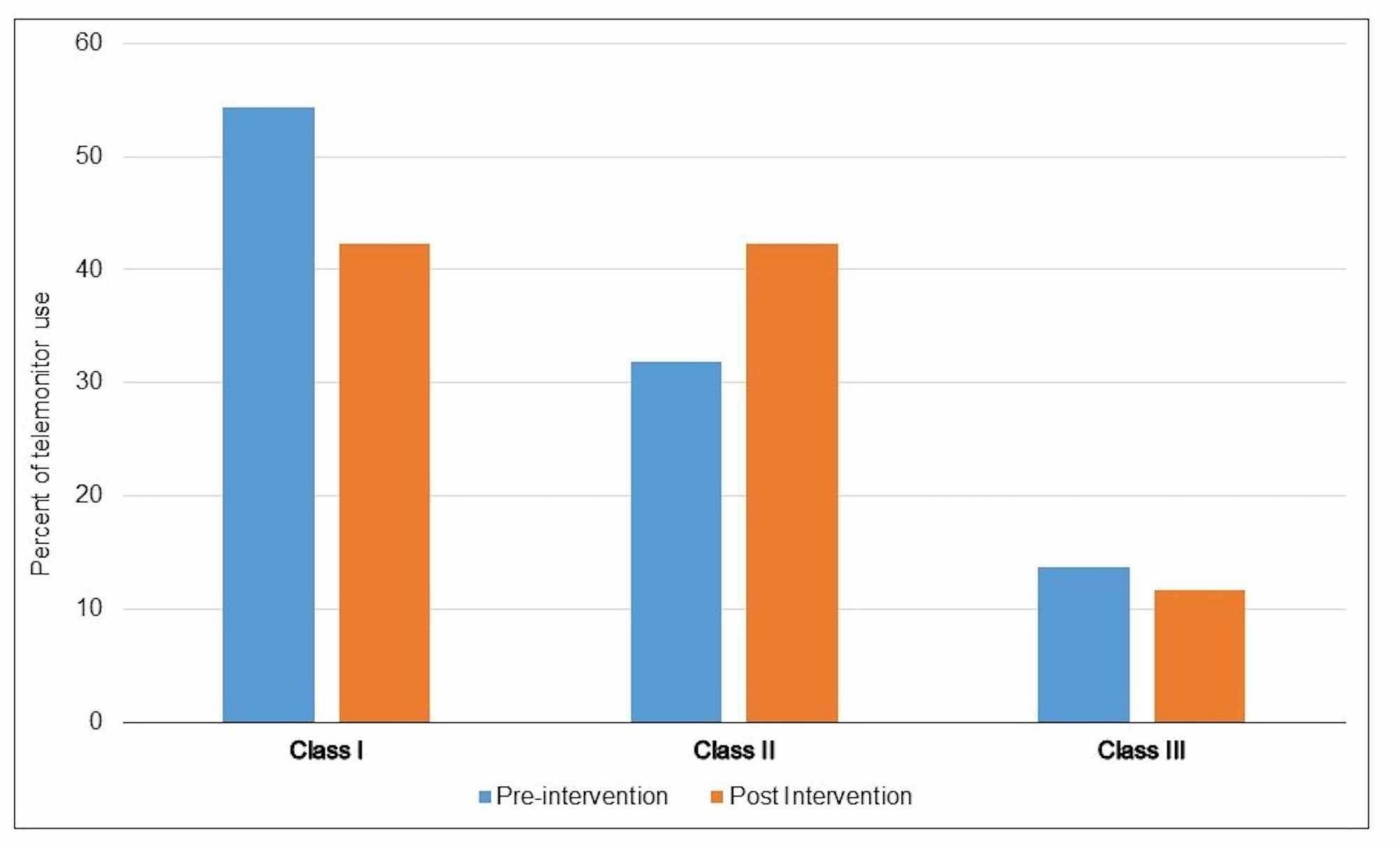
Use of telemonitor according to AHA indication (Class I: indicated, Class II: beneficial, and Class III: not indicated). AHA: American Heart Association

**Figure 3:**
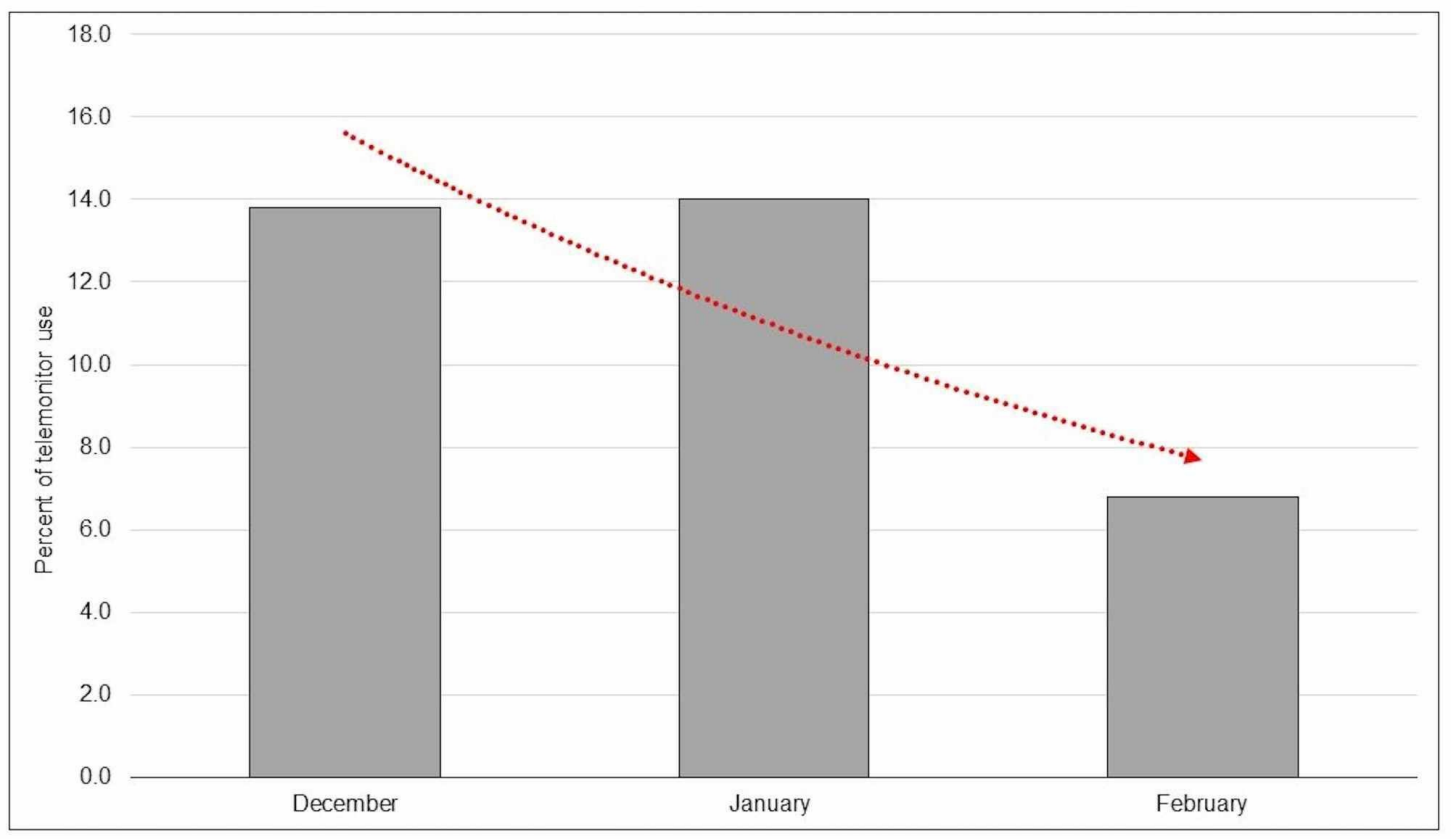
Declining trend of AHA class III (not indicated) use of telemetry in subsequent months in post-intervention period (trend line in red). AHA: American Heart Association

**Figure 4:**
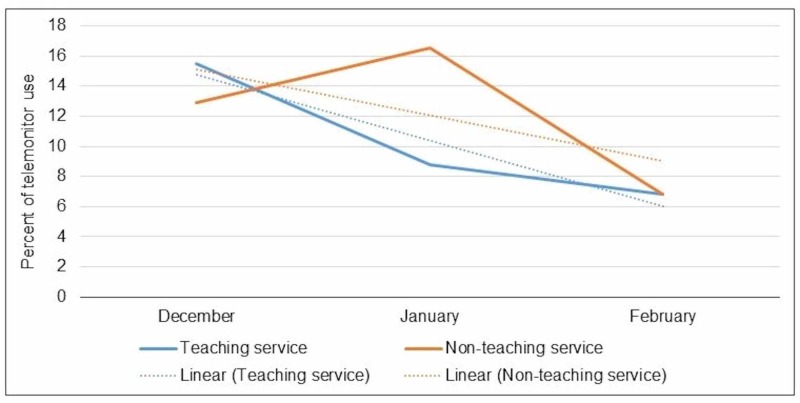
Trend of inappropriate use (AHA class III, not indicated) of telemonitor in teaching and non-teaching service. AHA: American Heart Association

In the pre-intervention group, 54% had class I (indicated), 32% had class II (beneficial), and 14% had class III (not indicated) indications. In post-intervention group, 46% had class I, 42% had class II, and 12% had class III indications. As such there was a 2% drop in the inappropriate use (Class III), but this was not statistically significant (Figure 2). The educational intervention resulted in a trend towards less inappropriate ordering of telemetry, particularly in teaching service over successive months (Figure 3). This effect was more pronounced in the teaching service which involved residents as compared to non-teaching service (Figure 4).

Telemonitor events

**Figure 5:**
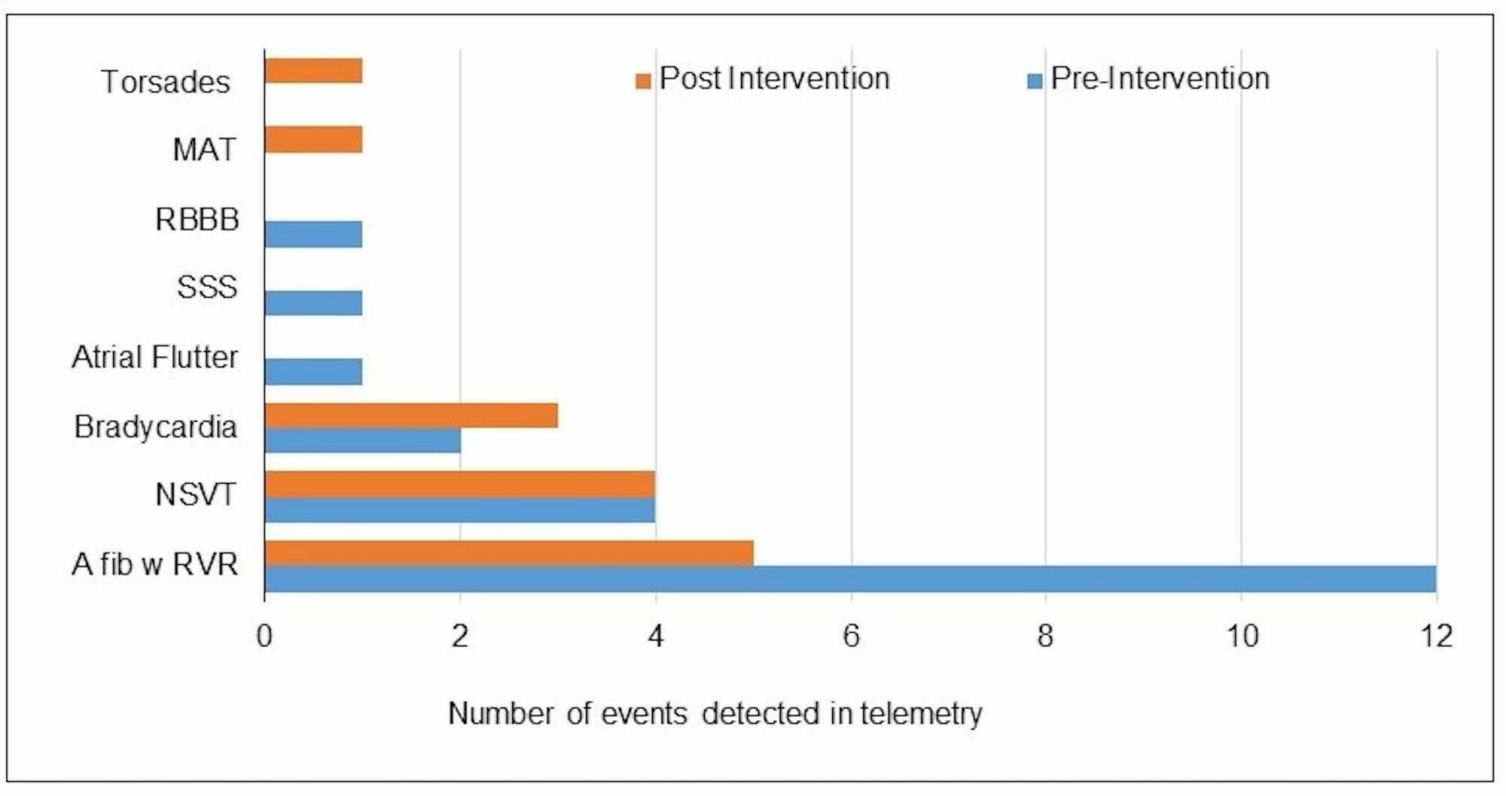
Cardiac events detected in the telemonitor. MAT: Multifocal atrial tachycardia; RBBB: Right bundle branch block; SSS: Sick sinus syndrome; NSVT: Non-sustained ventricular tachycardia; A fib w RVR: Atrial fibrillation with rapid ventricular response.

In the pre-intervention group, 22 events (7% of the sample) were detected on the telemonitor. Of these, actions were taken on six events (27%). In the post intervention group, there were 13 events (4%), and actions were taken on four events (27%). Actions included changes, or starting new medications, cardioversion or transfer to the intensive care unit (ICU). The commonest events picked up on the telemonitor were atrial fibrillation, non-sustained ventricular tachycardia, atrial flutter, bradycardia, torsade de pointes, and multifocal atrial tachycardia (Figure 5). Two patients died in the pre-intervention group and one in the post-intervention group from non-cardiac causes.

Reason for inappropriate telemetry use

**Figure 6:**
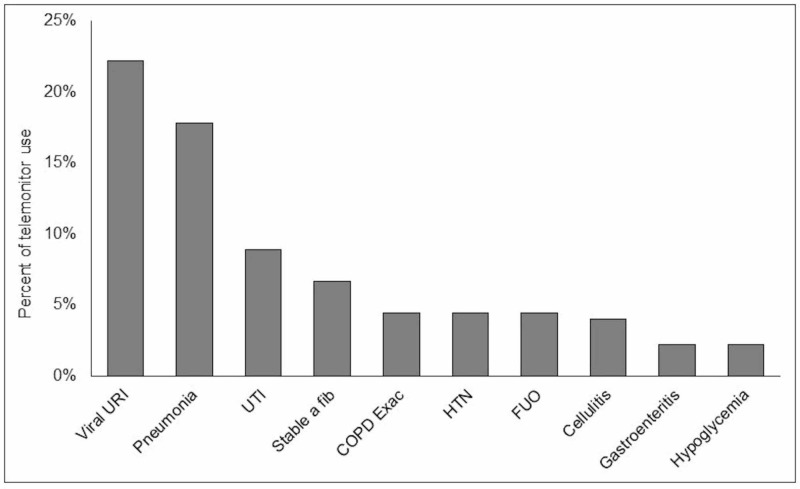
Common reasons for inappropriate use of telemonitor (AHA class III). URI: Urinary tract infection; a fib: Atrial fibrillation; COPD exac: Chronic obstructive pulmonary disease exacerbation; HTN: Hypertension; FUO: Fever of unknown origin; AHA: American Heart Association.

Viral respiratory infections accounted for the largest share of inappropriate telemetry use (22%). Other reasons included pneumonia (10%), urinary tract infections (9%), chronic stable atrial fibrillation (7%), COPD exacerbation, hypertension, fever of unknown origin and cellulitis which individually accounted for 4% (Figure 6).

## Discussion

This resident-run quality improvement project highlighted a previously noted problem in the provision of telemetry use when it is not clinically indicated. The Society for Hospital Medicine has made reducing the unnecessary use of telemetry a top priority in the Choosing Wisely campaign. Our study showed inappropriate utilization of telemonitor ranges between 12%-14%. This at first glance may seem reasonable but is offset by the significant cost associated with this resource. Inappropriate telemetry use rate in our study is lower than those noted in other hospitals of similar size where it was as high as 20-30% [6]. The use of guideline-based interventions has shown promise in reducing the inappropriate use of telemetry [7]. This study’s design to use guidelines to order telemonitor showed some modest improvement in the appropriateness of telemonitor use, particularly in patients on the teaching service. It seems that involvement of residents in the intervention process was associated with greater improvement. The probable reasons for this improvement include increased enthusiasm in the project run by fellow residents, and keenness to learn new information and use it appropriately.

One key finding was that most patients who were admitted to the telemetry unit tended to stay on cardiac monitor regardless of resolution of the indication or having no indication at the outset. More than 90% of patients remained on telemetry throughout their admission. This signifies that once a patient is diagnosed with possible cardiac or neurological event, they are likely to be monitored with telemonitor throughout their hospitalization. The educational intervention did not result in a significant change in the telemonitor discontinuation rates. Further interventions likely need to be incorporated to remind providers of the need to discontinue telemetry at the earliest appropriate opportunity as clinically indicated. A study by Rizvi et al. showed that incorporating pop-ups in the electronic medical system for discontinuing telemetry reduced overuse of telemetry by 37% [8]. Such an intervention or the use of expiration dates with initial order of telemetry, which currently does not exist in our hospital, may be helpful in stopping telemetry when no longer indicated.

This study further illustrated that only few events were detected on telemetry, and an even smaller number of these events required change of management. Clinically significant events were only noted in 3-7% of patients and only a third of these events resulted in changes in medications or transfer to a higher level of care such as transfer to the intensive care unit. This low yield from telemetry and rare subsequent transfer to ICU has been documented in other studies [9-12]. Our results also showed the arrhythmias were detected only in the patients who had appropriate indication for use of telemetry, further reinforcing the need to restrict telemetry use in clinical- and guideline-directed indicated cases. Further exploration into incorporating an electronic order system, where providers indicate the reason for ordering telemetry, can result in further improvement in the ordering pattern. It appears that addressing this problem will likely require a multi-pronged approach, incorporating a sustained educational intervention, and indication-based electronic order system.

Analysis of the main reasons for inappropriate telemetry use showed that a significant proportion of patients had telemetry ordered for respiratory infections, chronic stable atrial fibrillation, urinary tract infections and exacerbations of obstructive lung disease. The median age of patients in our study was 77 years, and thus the concern for sick elderly patients likely prompted admission for closer observation with use of telemonitor. However, the yield of telemonitor use is typically low in such patients. A study of telemetry on geriatric patients with low risk for in-hospital coronary events showed that none of the patients admitted to telemetry developed clinically significant events [13].

Our study was limited in that the duration of post intervention evaluation period was short. Three months was likely not adequate to evaluate the sustained effect of the intervention. Determination of the appropriateness of telemetry use was based on what was documented in the charts. It is possible that the ordering providers may have had other reasons unclear to the chart reviewer(s) which possibly could have created a bias in the result. The sample size was also relatively small and possibly could have overestimated or underestimated the outcomes. Moreover, the retrospective nature of the study is vulnerable to selection bias. One of the limitations is that the study population was not homogeneous between pre- and post-intervention group. As a result, for class II indication, there was 105 (32%) patients in pre-intervention group and 162 (42%) patients in post-intervention group. This difference in sample size in two groups was significant, which might have affected the interpretation of data for class II indication. Nonetheless, this was a resident-run quality improvement project to increase awareness among house staffs as well as to improve guideline and evidence-based use of telemonitor in hospitalized patients.

## Conclusions

Our results highlight that emphasizing guideline-based utilization of the telemonitor in non-ICU patients can aid in ensuring appropriate use of this resource. However, the effect of the educational campaign was modest and further studies with longer post-intervention follow-up and combining with electronic medical system interventions need to be assessed to see greater changes in ordering patterns of providers.
